# Desmoid fibromatosis in a male breast with gynecomastia: A case report

**DOI:** 10.1016/j.radcr.2023.09.104

**Published:** 2023-10-27

**Authors:** Sakina Moussaddykine, Marieme Sy N'deye

**Affiliations:** Senology Department, Université libre de Bruxelles, Erasme Hospital, Route de Lennik 808 1070 Anderlecht, Belgium

**Keywords:** Breast, Desmoid fibromatosis, Male, MRI, Ultrasound, X-ray

## Abstract

We present a unique case of a male patient with a history of gynecomastia, who sought medical evaluation due to the discovery of a palpable left breast mass. Mammography revealed a spiculated mass in the upper quadrant of the left breast at 10-o'clock, and subsequent ultrasound and MRI confirmed its presence. Ultrasound-guided biopsy identified the mass as desmoid fibromatosis. This case highlights the diagnostic challenge posed by desmoid fibromatosis, especially in men, as it can mimic breast cancer both clinically and radiologically. Desmoid fibromatosis, although locally invasive, does not metastasize and necessitates extensive resection due to a high recurrence rate.

## Introduction

Desmoid fibromatosis of the breast is a rare condition that primarily affects women in their *40s* and *50s*, with even fewer reported cases in men. According to the literature, it represents a mere 0.2% of all breast tumors [1]. Only a limited number of cases have been documented in existing literature, with our knowledge extending to just 9 reported instances of desmoid fibromatosis in the male breast [[2], [3], [4], [5], [6], [7], [8], [9]].

This pathology exhibits similar characteristics to breast carcinoma when assessed through various imaging modalities. Our case report contributes an additional layer to the existing body of literature by highlighting the diagnostic challenges posed by this rare condition.

## Description of the case report

We report a case of a 40-year-old man presenting to the gynecology consultation for self-palpation of a left breast mass for 4 months in a context of known chronic bilateral gynecomastia. The patient was known to have insulin-treated, well-balanced diabetes, with no family or personal history of breast cancer.

Clinical examination confirmed the presence of a tumor measuring 1-2 cm in the upper quadrant of the left breast at 10-o'clock, mobile with respect to the skin.

The patient was referred to the medical imaging department where a bilateral mammogram was performed. Two external-oblique views were performed. It revealed a spiculated mass in the upper quadrant of the left breast at 10-o'clock, measuring 14 × 16 × 19 mm, in contact with the pectoralis major muscle. These images were compared to a previous mammogram performed 2 years earlier, showing an increase in the bilateral gynecomastia on the one hand and the development of this single mass in the left breast on the other.

**Fig. 1:**
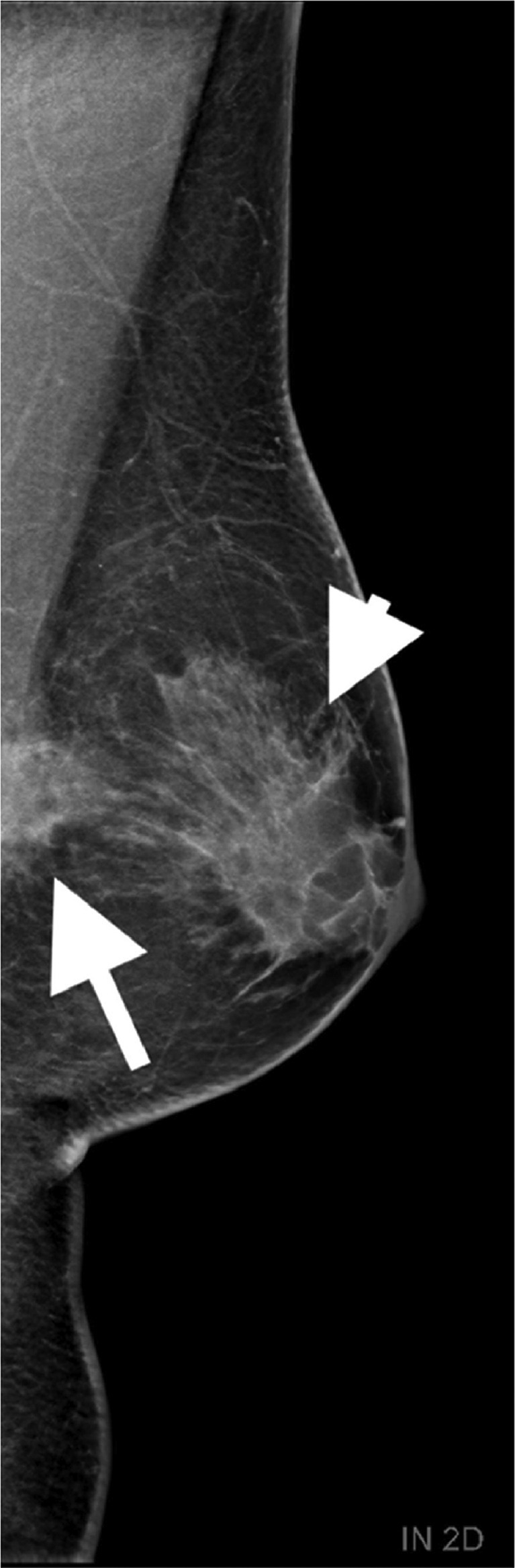
Mammogram of the left breast. Depicting gynecomastia (white arrowhead) and the emergence of a mass with spiculated contours (white arrow) in direct contact with the pectoral muscle, situated in the posterior region of the breast.

These criteria led us to classify the mass as Breast Imaging-Reporting And Data System (BI-RADS) 5 (Figs. 1 A and B).

**Fig. 2:**
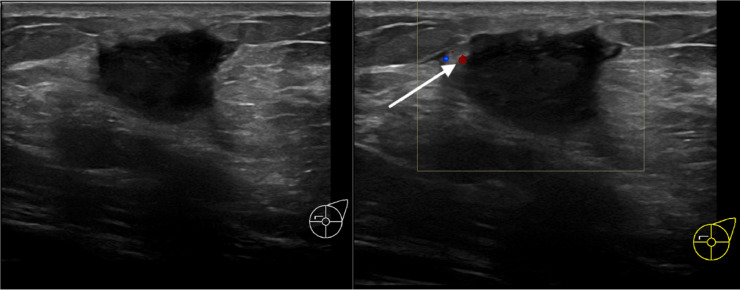
Ultrasound of the left breast with transversal orientation. Displays a hypoechoic mass in the in the upper quadrant at 10-o'clock with indistinct contours and posterior shadowing. Notably, there is a few color Doppler vascularization (white arrow).

An ultrasound scan revealed a hypoechoic mass with indistinct, lobulated contours located in the upper quadrant of the left breast at 10-o'clock. The assessment of its relationship with the pectoral muscle was challenging due to the posterior shadowing associated with the mass and its deep location. No axillary adenopathy was found (Fig. 2).

**Fig. 3:**
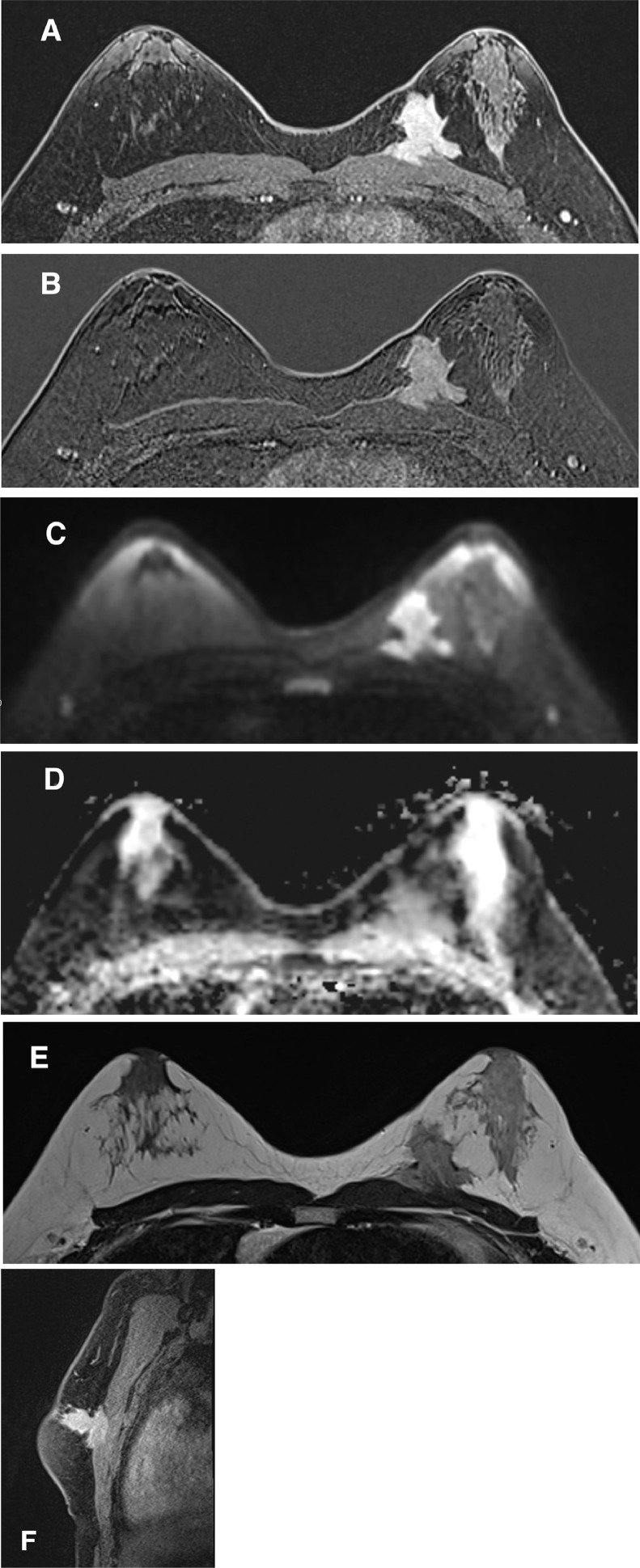
MRI (A) Dynamic T1-weighted axial sequence. (B) Dynamic T1-weighted axial sequence with subtraction. (C) Axial diffusion sequence. (D) ADC axial sequence. (E) Axial T2-weighted sequence. (F) Late T2-weighted sagittal sequence. Depicts a spiculated mass in the upper quadrant at 10-o'clock of the left breast, demonstrating enhancement after contrast injection, accompanied by skin retraction and infiltration of the pectoralis major muscle. Additionally, diffusion restriction and late enhancement are observed.

To assess the anatomical relationship of this mass, breast magnetic resonance imaging (MRI) was performed. The mass measured 39 mm in the cephalocaudal axis, 37 mm in the anteroposterior axis, and 41 mm in the laterolateral axis. It was located in the upper quadrant of the left breast at 10-o'clock and exhibited features suspicious of malignancy, as described on both modalities. Furthermore, it was associated with skin retraction and infiltration of the pectoralis major muscle (Fig. 3).

Following these findings, 4 tissue samples were obtained under ultrasound guidance. The anatomopathological results supported the diagnosis of desmoid fibromatosis.

A thoraco-abdominal CT scan revealed no evidence of distant metastases. PET-CT results indicated the absence of metabolic evidence for metastatic bone lesions.

After a multidisciplinary oncological consultation, the decision was made to proceed with a left mastectomy.

Genetic analysis of the mastectomy specimen identified a mutation in the CTNNB1 gene at position T41A, while no mutation was detected in the APC gene.

## Discussion

Desmoid fibromatosis, or desmoid tumor, is a mesenchymal tumor arising from the proliferation of fibroblastic and myofibroblastic cells originating from the muscle fascia. The precise cause remains unknown, although a history of trauma or surgery may be considered as potential triggers.

A notable characteristic of desmoid tumors is their propensity for local recurrence, with rates ranging from 21% to 23%, depending on the case [11]. Consequently, treatment recommendations vary between genders. In women, enlarged tumorectomy with generous resection margins is advocated for aesthetic reasons and to mitigate the risk of local recurrence. In cases of recurrence, mastectomy becomes a consideration. Conversely, in men, mastectomy is typically recommended as the initial course of action, as seen in our patient's case.

An association between desmoid fibromatosis and Gardner syndrome, a variant of familial adenomatous polyposis characterized by the presence of osteomas and epidermoid cysts, has been established [12]. While there is only one documented case report in the literature of a desmoid tumor in the male breast within the context of Gardner syndrome [10], colonoscopy is recommended for the screening of colonic polyps associated with this syndrome.

Desmoid tumors share malignant characteristics with breast carcinoma, rendering imaging incapable of distinguishing between the two. Therefore, biopsy is the definitive diagnostic tool.

Pathologically, these tumors are characterized by the proliferation of slender fibroblastic cells, a collagen-rich stroma, prominent vascularization, and entrapped atrophic skeletal muscle fibers, which can mimic giant cells at the periphery of the lesion. Mutations in exon 3 of the CTNNB1 gene, ranging from 84% to 87%, are frequently observed in sporadic desmoid tumors [13].

In addition to classical surgical therapy, alternative treatments have been proposed in the literature, including radiotherapy, chemotherapy, and anti-inflammatory drugs. These alternatives may be considered for patients with unresectable tumors or those requiring extensive resection [7].

While consensus on post-treatment follow-up is lacking in previous studies, it is prudent to recommend follow-up due to the recurrence potential within the 3-year timeframe. Some authors propose annual MRI follow-up [1], while others suggest ultrasound assessments at 6 months and 1-year postsurgery over a 3-year period [4].

## Conclusion

Desmoid fibromatosis of the male breast is a rare entity with imaging criteria that mimic breast neoplasia, posing a diagnostic challenge. The final diagnosis requires pathological analysis.

Due to its rarity, surgical management is crucial. In women, an enlarged tumorectomy with wide resection margins is favored, while in men, preferential mastectomy is recommended due to aesthetic considerations.

## Patient consent

I declare that written, informed consent for publication of the case was obtained from the patient.
